# Evolutionary Trends of A(H1N1) Influenza Virus Hemagglutinin Since 1918

**DOI:** 10.1371/journal.pone.0007789

**Published:** 2009-11-17

**Authors:** Jun Shen, Jianpeng Ma, Qinghua Wang

**Affiliations:** 1 Department of Bioengineering, Rice University, Houston, Texas, United States of America; 2 Department of Biochemistry and Molecular Biology, Baylor College of Medicine, Houston, Texas, United States of America; Institute of Infectious Disease and Molecular Medicine, South Africa

## Abstract

The Pandemic (H1N1) 2009 is spreading to numerous countries and causing many human deaths. Although the symptoms in humans are mild at present, fears are that further mutations in the virus could lead to a potentially more dangerous outbreak in subsequent months. As the primary immunity-eliciting antigen, hemagglutinin (HA) is the major agent for host-driven antigenic drift in A(H3N2) virus. However, whether and how the evolution of HA is influenced by existing immunity is poorly understood for A(H1N1). Here, by analyzing hundreds of A(H1N1) HA sequences since 1918, we show the first evidence that host selections are indeed present in A(H1N1) HAs. Among a subgroup of human A(H1N1) HAs between 1918∼2008, we found strong diversifying (positive) selection at HA_1_ 156 and 190. We also analyzed the evolutionary trends at HA_1_ 190 and 225 that are critical determinants for receptor-binding specificity of A(H1N1) HA. Different A(H1N1) viruses appeared to favor one of these two sites in host-driven antigenic drift: epidemic A(H1N1) HAs favor HA_1_ 190 while the 1918 pandemic and swine HAs favor HA_1_ 225. Thus, our results highlight the urgency to understand the interplay between antigenic drift and receptor binding in HA evolution, and provide molecular signatures for monitoring future antigenically drifted 2009 pandemic and seasonal A(H1N1) influenza viruses.

## Introduction

Since April 2009, a global outbreak caused by the swine-origin 2009 A(H1N1) influenza virus has spread to numerous countries [1], [2], [3], [4], [5], [6], [7], [8], [9], [10], which warranted the declaration of “Pandemic (H1N1) 2009” by the World Health Organization on June 11, 2009. As of September 6, there had been over 277,607 infected individuals and at least 3,205 confirmed human deaths worldwide.

The Pandemic (H1N1) 2009 is not the first human pandemic caused by A(H1N1) influenza virus. During 1918∼1919, the “Spanish” A(H1N1) influenza virus swept across the globe, infected ∼25% of the entire population and claimed at least 50 million human lives worldwide [11]. In subsequent years, A(H1N1) influenza virus continued to circulate among humans and caused a number of severe outbreaks between 1920s and 1950s [12], [13], [14], [15], [16], [17], [18], [19], [20], in particular the A(H1N1) epidemic in 1950∼1951 with mortality exceeding those of the 1957 “Asian” and 1968 “Hong Kong” pandemics [18], [19], [20]. In 1957, A(H1N1) influenza virus disappeared, replaced by a reassorted A(H2N2) influenza virus [21]. However, the A(H1N1) influenza virus reappeared in 1977, with a close genetic and antigenic similarity to those A(H1N1) viruses isolated in 1950 [22], [23], [24], and has co-circulated with A(H3N2) and type B influenza virus to cause seasonal human epidemics ever since.

The same 1918 pandemic A(H1N1) influenza virus was also spread to swine during 1918∼1919, and became the so-called “classical” swine influenza [8], [25], [26], [27], first isolated in North American in 1930 [26] and in Europe in 1976 [28], [29]. In 1979, a novel lineage of avian-like A(H1N1) influenza virus, believed to have derived from closely related Eurasia avian influenza viruses, emerged in swine in Europe [30] and replaced the classical swine A(H1N1) virus in this region [31], [32], [33]. These two classes of swine A(H1N1) viruses displayed different evolutionary trajectories [34]. In1998, a new triple-reassortant A(H3N2) virus, derived from North American avian, classical swine A(H1N1) and human A(H3N2) viruses, caused outbreaks in North American swine [35], [36]. Mixing of the triple-reassortant H3N2 with established swine lineages gave rise to H1N1 and H1N2 reassortant swine viruses [37], [38]. Since 2007, human infection caused by A(H1N1) swine virus has become a health concern in the United States [7].

The 2009 A(H1N1) influenza virus has its origin as a reassortant from a Eurasian avian-like swine A(H1N1) virus and a triple-reassortant virus circulating in North American swine [1], [2], [3], [4], [5], [6], [7], [8]. As such, the 2009 A(H1N1) virus contains NA and M from Eurasian avian-like swine A(H1N1) virus, and the remaining genes from the triple-reassortant virus - PB2 and PA (avian virus), PB1 (human A(H3N2)), and HA, NP and NS (classical swine A(H1N1)) [1], [2], [3], [4], [5], [6], [7], [8]. In a sense, we are continuingly living in a pandemic that started in 1918 [27]. Thus, it is not surprising for the similarly mild first waves of the 1918 and 2009 pandemics. Notably, the second wave of the “Spanish” influenza in the fall of 1918 became much more lethal, peaked within one month of the initial introductions in many communities [11]. This makes influenza virologists and healthcare officials fear that further mutations in the 2009 A(H1N1) virus could also lead to a potentially more dangerous second wave in subsequent months. Thus, in-depth studies on the 1918 pandemic strains as well as their post-pandemic decedents should provide critical new insights into the evolution of A(H1N1) in general, and the pandemic potential of the 2009 A(H1N1) in particular.

HA is one of the two major glycoproteins on the surface of influenza virus. It is the primary antigen that elicits host immune response, and is also responsible for binding to sialic-acid receptors and for mediating viral entry into host cells [39]. The hallmarks of highly pathogenic influenza viruses among human population include easy human-to-human transmission as a result of high affinity of HA for human-like α(2,6) receptors, and significant difference in sequence and antigenicity of HA with existing seasonal and vaccine strains [1], [2], [39]. It has been demonstrated on 1918 A(H1N1) HA that HA_1_ D190 and D225 are key determinants for effective binding to human-like α(2,6) receptors and consequently high infectivity of the virus among human population [11], [40], [41], [42]. A single mutation D225G reduced the binding affinity for α(2,6) receptors [41], [42] and the infectivity of the virus [40], while a double variant D190E/D225G rendered the HA non-binding to α(2,6) receptors [41], [42] and the virus non-infectious [40].

In A(H3N2) virus, HA is the major agent for host-driven antigenic drift [43], [44]. However, it is unclear whether or not and, if yes, how human immunity imposes selection on A(H1N1) HA. In order to address this critical issue, we undertook a systematic computational analysis of the evolution of H1 HA in the region of HA_1_, which is the primary target for host immunity selection [43].

Recent years have witnessed an explosive expansion of available computational methods for phylogenetic analysis of selective pressure, including a variety of methods that look for different types of positive selection such as diversifying selection, toggling selection and directional selection [45], [46], [47], [48], [49], [50], [51], [52], [53], [54] implemented in software packages such as HyPhy [55], MrBayes [56], [57] and PAML [45]. Here we used PAML 4.0 [45] for calculation of heterogeneous selection pressure at each codon and HyPhy [55] for directional selection in 335 non-egg-adapted and 32 egg-adapted human A(H1N1) HA sequences. These sequences were from A(H1N1) viruses isolated all around the globe between 1918∼2009. In addition, we also analyzed 42 classical swine A(H1N1) HA sequences for their close relationship to the 2009 A(H1N1) HA.

In PAML 4.0 [45], a number of models are available: the branch models allow the ω ratio to vary among branches in the phylogenetic tree and can be used to detect positive selection on particular branches [46], [58]; the site models allow the ω ratio to vary among sites and can be used to detect positive selection at particular sites [59], [60]; the branch-site models allow the ω ratio to vary both among sites and among branches [61] and can be used to detect positive selection that affects only a few sites in a few branches.

In this analysis, a large dataset composed of over 300 sequences was used to ensure sufficient representative sequences for the total time span of 91 years, which made it impractical for the use of branch-site models in our calculations. However, by separating the sequences into distinct subgroups based on their phylogenetic relationship and applying the site models in PAML 4.0 [45], we successfully detected the branch and the specific sites therein that were under host-driven positive selection. Our study revealed differential evolutionary trends of A(H1N1) HA since 1918, which provided molecular signatures for monitoring future antigenically drifted 2009 pandemic and seasonal A(H1N1) influenza viruses.

## Results and Discussion

### Phylogenetic Analysis of Human A(H1N1) HA Sequences Since 1918

It is known that egg-adapted influenza viruses tend to have non-natural host-associated modifications at certain sites of HA sequences [62], [63], [64]. To eliminate the effects of such modifications in our analysis, we selected only 333 HA sequences of A(H1N1) viruses between 1918∼2009 (as of July 10, 2009) with a well-documented record that they had never been passaged in chicken eggs at any stage. Furthermore, intragenic recombination may give rise to false positives in subsequent detection of positively selected codons [65], thus the Recombination Detection Program (RDP3) [66] was used to make sure that all HA sequences used in this study were free of recombination, agreeing with previous observations that intragenic recombination is rare for HA [67]. The nucleotide sequences of 333 A(H1N1) HAs in the region of HA_1_ including the signal peptide, were analyzed by the ClustalW method [68]. The phylogeny tree suggested that these HA sequences belong to two major groups: the majority of HA sequences from 1918 to 2008 formed group I, and those of the 2009 A(H1N1) together with a strain isolated in 2007 formed group II (Fig. S1). The separation of the 2009 A(H1N1) HAs from HAs of established human A(H1N1) viruses between 1918∼2008, including the 1918 pandemic and the seasonal A(H1N1) viruses, was consistent with the proposed swine origin of HAs in these viruses [1], [2], [3], [4], [5], [6], [7], [8]. The low sequence identity (∼73%) between the 2009 A(H1N1) HA with seasonal and vaccine A(H1N1) HAs might explain why people were in general immunologically naïve to the former [8], [69]. In fact, there did not exist cross-reactivity between the 2009 and seasonal A(H1N1) viruses [8], nor did the vaccination with recent (2005∼2009) annual vaccines provide immune protection against the 2009 A(H1N1) virus [69].

### Evidence for Host-Driven Antigenic Drift in Human A(H1N1) HAs

In order to understand whether host-driven antigenic drift is imposed on the evolution of HA_1_ of A(H1N1) virus, we used likelihood ratio tests (LRT) in the software package PAML 4.0 [45] to identify the presence or absence of *positive selection*. In this context, positive selection referred to a significant excess of amino-acid altering (non-synonymous) substitutions over silent (synonymous) substitutions in nucleotide sequences. Large LRT values (or small *p*-values) between alternative models and null models, such as M2a vs. M1a, M8 vs. M7, or M8 vs. M8a, led to the rejection of the null models.

Since HA sequences of group I was further divided into five subgroups (Fig. S1), the PAML calculation was carried out on each of these five subgroups and on group II (Table 1). For group I-*i* that included three 1918 pandemic A(H1N1) HAs, in order to increase the sample size, we also included two partial sequences, A/London/1/1918 and A/London/1/1919 [11]. Except for the subgroup I-*v*, all other subgroups of group I had very low LRT values and large *p*-values (Table 1, 2), indicating predominantly neutral or purifying selection. These results were consistent with the overall low prevalence of A(H1N1) virus during the period of 1979∼2006 [70], and agreed well with a previous study that focused on 1995∼2005 A(H1N1) isolates where no positive selection was detected [71]. In sharp contrast, group I-*v* 2006∼2008 had ω>10 and LRT>60, which provided strong evidence for positive selection (Table 1, 2) and agreed with the necessity to update the A(H1N1) vaccine strain using A/Brisbane/59/07 for the 2008∼2009 season. Group II including 73 HAs of 2009 A(H1N1) and one of 2007 A(H1N1) also had a very low LRT rate ratio (Table 1, 2). Given the largely nonexistence of human immunity against the 2009 A(H1N1), the lack of positive selection among group II was expected. However, with more mild infections rapidly propagating among human population in the first wave, the gradually established human immunity might drive positive selection in future isolates of 2009 A(H1N1) strains.

**Table 1:** The values of log-likelihood (l), *d*_N_/*d*_S_, and parameter estimates in CODEML analysis of human A(H1N1) Has.

Model	l	*d*_N_/*d*_S_	Parameters estimates
**I-** ***i*** ** 1918∼1919 (5 strains)** 1			
M0 (one-ratio)	−806.78	0.516	ω = 0.516
M1a (nearly neutral)	−805.91	0.323	*p*_0_ = 0.677 (*p*_1_ = 0.323), ω_0_ = 0 (ω_1_ = 1)
M2a (positive selection)	−804.19	0.564	*p*_0_ = 0.963, *p*_1_ = 0 (*p*_2_ = 0.037), ω_0_ = 0 (ω_1_ = 1), ω_2_ = 15.421
M7 (beta)	−805.92	0.300	*p* = 0.005, *q* = 0.012
M8a (beta&ω = 1)	−805.91	0.323	*p*_0_ = 0.846 (*p*_1_ = 0.154), *p* = 0.005, *q* = 0.020, ω_s_ = 1
M8 (beta&ω>1)	−804.19	0.561	*p*_0_ = 0.963 (*p*_1_ = 0.037), *p* = 0.005, *q* = 7.228, ω_s_ = 15.242
**I-** ***ii*** ** 1979∼2000 (45 strains)^2^**			
M0 (one-ratio)	−2489.23	0.223	ω = 0.223
M1a (nearly neutral)	−2486.25	0.242	*p*_0_ = 0.855 (*p*_1_ = 0.145), ω_0_ = 0.113 (ω_1_ = 1)
M2a (positive selection)	−2486.25	0.242	*p*_0_ = 0.855, *p*_1_ = 0.056 (*p*_2_ = 0.089), ω_0_ = 0.113 (ω_1_ = 1), ω_2_ = 1
M7 (beta)	−2485.63	0.232	*p* = 0.327, *q* = 1.074
M8a (beta&ω = 1)	−2485.63	0.232	*p*_0_ = 1 (*p*_1_ = 0), *p* = 0.327, *q* = 1.074, ω_s_ = 1
M8 (beta&ω>1)	−2485.63	0.232	*p*_0_ = 1 (*p*_1_ = 0), *p* = 0.327, *q* = 1.074, ω_s_ = 1
**I-** ***iii*** ** 2000∼2001 (22 strains)^2^**			
M0 (one-ratio)	−1686.21	0.279	ω = 0.279
M1a (nearly neutral)	−1684.58	0.261	*p*_0_ = 0.793 (*p*_1_ = 0.207), ω_0_ = 0.068 (ω_1_ = 1)
M2a (positive selection)	−1683.09	0.287	*p*_0_ = 0.994, *p*_1_ = 0 (*p*_2_ = 0.006), ω_0_ = 0.225 (ω_1_ = 1), ω_2_ = 10.636
M7 (beta)	−1684.52	0.265	*p* = 0.046, *q* = 0.127
M8a (beta&ω = 1)	−1684.58	0.261	*p*_0_ = 0.793 (*p*_1_ = 0.207), *p* = 7.211, *q* = 98.93, ω_s_ = 1
M8 (beta&ω>1)	−1683.10	0.287	*p*_0_ = 0.994 (*p*_1_ = 0.006), *p* = 28.774, *q* = 99, ω_s_ = 10.638
**I-** ***iv*** ** 2001∼2007 (89 strains)^2^**			
M0 (one-ratio)	−2720.83	0.187	ω = 0.187
M1a (nearly neutral)	−2709.20	0.181	*p*_0_ = 0.883 (*p*_1_ = 0.117), ω_0_ = 0.072 (ω_1_ = 1)
M2a (positive selection)	−2708.27	0.187	*p*_0_ = 0.906, *p*_1_ = 0.076 (*p*_2_ = 0.018), ω_0_ = 0.085 (ω_1_ = 1), ω_2_ = 1.915
M7 (beta)	−2708.99	0.183	*p* = 0.137, *q* = 0.612
M8a (beta&ω = 1)	−2709.40	0.180	*p*_0_ = 0.885 (*p*_1_ = 0.115), *p* = 7.871, *q* = 98.995, ω_s_ = 1
M8 (beta&ω>1)	−2708.82	0.186	*p*_0_ = 0.973 (*p*_1_ = 0.027), *p* = 0.375, *q* = 2.298, ω_s_ = 1.936
**I-** ***v*** ** 2006∼2008 (100 strains)^2^**			
M0 (one-ratio)	−3871.33	0.303	ω = 0.303
M1a (nearly neutral)	−3813.28	0.241	*p*_0_ = 0.828 (*p*_1_ = 0.172), ω_0_ = 0.082 (ω_1_ = 1)
M2a (positive selection)	−3782.07	0.313	*p*_0_ = 0.807, *p*_1_ = 0.188 (*p*_2_ = 0.005), ω_0_ = 0.083 (ω_1_ = 1), ω_2_ = 11.142
M7 (beta)	−3814.21	0.246	*p* = 0.139, *q* = 0.427
M8a (beta&ω = 1)	−3812.40	0.231	*p*_0_ = 0.862 (*p*_1_ = 0.138), *p* = 0.497, *q* = 3.969, ω_s_ = 1
M8 (beta&ω>1)	−3781.55	0.305	*p*_0_ = 0.994 (*p*_1_ = 0.006), *p* = 0.180, *q* = 0.546, ω_s_ = 10.554
**II 2007∼2009 (74 strains)^2^**			
M0 (one-ratio)	−2374.98	0.277	ω = 0.277
M1a (nearly neutral)	−2373.20	0.282	*p*_0_ = 0.922 (*p*_1_ = 0.078), ω_0_ = 0.222 (ω_1_ = 1)
M2a (positive selection)	−2372.81	0.282	*p*_0_ = 0.922, *p*_1_ = 0.035 (*p*_2_ = 0.042), ω_0_ = 0.222 (ω_1_ = 1), ω_2_ = 1
M7 (beta)	−2373.21	0.281	*p* = 1.218, *q* = 3.079
M8a (beta&ω = 1)	−2374.03	0.282	*p*_0_ = 0.945 (*p*_1_ = 0.055), *p* = 3.243, *q* = 10.201, ω_s_ = 1
M8 (beta&ω>1)	−2372.81	0.282	*p*_0_ = 0.946 (*p*_1_ = 0.054), *p* = 3.158, *q* = 9.900, ω_s_ = 1

1 Due to the inclusion of two partial sequences of A/London/1/1918 and A/London/1/1919 in this subgroup, the analysis was performed on a total of 187 amino-acid residues that covered the antigenic and receptor-binding sites in the region of HA_1_ (51∼237) [11]. ^2^ The analysis was performed on the first 340 residues of HA_1_ including the signal peptide.

**Table 2:** LRT tests for HA_1_ sequences of human A(H1N1) influenza viruses.

	LRT (M2a − M1a) (2Δl) (*p*-values)^1^	LRT (M8 − M7) (2Δl) (*p*-values)^1^	LRT (M8 − M8a) (2Δl) (*p*-values)^2^
I-*i* 1918∼1919 (5 strains)	3.44 (0.1791)	3.46 (0.1773)	3.44 (0.0318)
I-*ii* 1979∼2000 (45 strains)	0	0	0
I-*iii* 2000∼2001 (22 strains)	2.98 (0.2254)	2.84 (0.2417)	2.96 (0.0427)
I-*iv* 2001∼2007 (89 strains)	1.86 (0.3946)	0.34 (0.8437)	1.16 (0.1407)
I-*v* 2006∼2008 (100 strains)	62.42 (0.0000)	65.32 (0.0000)	61.70 (0.0000)
II 2007∼2009 (74 strains)	0.78 (0.6771)	0.80 (0.6703)	2.44 (0.0591)

1 We used the degree of freedom of 2 for these LRT tests that is expected to be too conservative. ^2^ The *p*-values were calculated from χ^2^ distribution using degree of freedom of 1 that was then divided by a factor of 2 for the mixture distribution, as suggested by the author of PAML 4.0.

### Identification of Positively Selected Codons in Human A(H1N1) HAs

**Figure 1:**
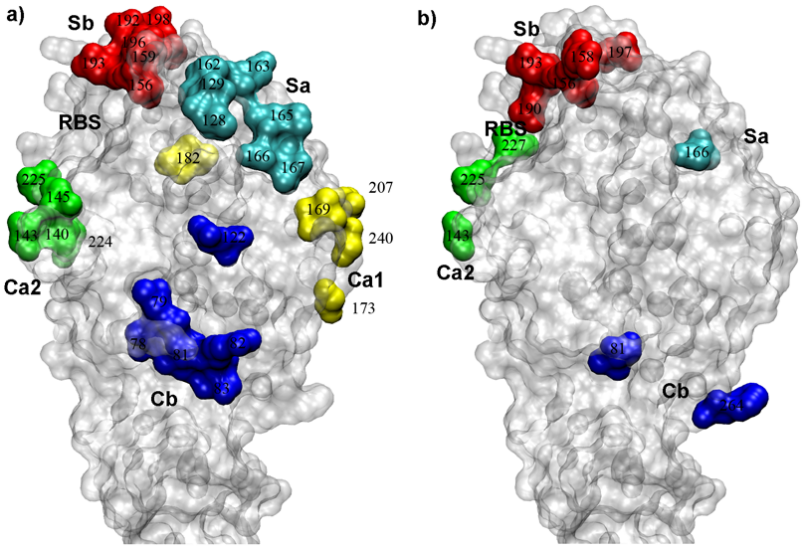
Antigenic structure and positive selection of A(H1N1) HA. **a**) Antigenic structure of A/PR/8/34 (H1N1) HA (PDB accession code 1RU7 [78]). Five antigenic sites were identified by using a large number of monoclonal antibodies [73], [74]: Sa (cyan), Sb (red), Ca1 (yellow), Ca2 (green), Cb (blue), using H3 HA numbering. The receptor-binding site (RBS) was labeled for reference. **b**) Codons on A(H1N1) HA that were identified to be under various selection in PAML and HyPhy analysis.

In order to understand how H1 HA sequences were positively selected by human existing immunity, the CODEML [72] program in PAML 4.0 was used on subgroup I-*v* in which about 0.6% codons were found to be under positive selection (Table 1, 2). Both M2a and M8 models identified HA_1_ 156 and 190 with greater than 95% posterior probabilities to be under positive selection (Table 3). In previous studies, the antigenic structure of H1 HA (A/PuertoRico/8/1934) had been determined to include five distinct antigenic sites on the globular domain: Sa, Sb, Ca1, Ca2 and Cb [73], [74] (Fig. 1a). Both of these positively selected codons were located on the site Sb (Fig. 1b). The focus of positive selection on the Sb antigenic site was consistent with a cross-reactivity analysis of various epidemic H1N1 strains using monoclonal antibodies that it was under much higher pressure for mutations [74].

**Table 3:** Codons under positive selection in HA_1_ of human A(H1N1) influenza viruses.

		Positively selected sites^1^
**I-** ***v*** ** 2006∼2008 (100 strains)**	M2a	156 (97.5%), 190 (100%)
	M8	156 (99.7%), 190 (100%)

1 Positively selected sites from PAML 4.0 [45] using Bayes Empirical Bayes analysis [72]. Only codons with greater than 95% posterior probabilities to be under positive selection were listed with the corresponding posterior probabilities shown in parentheses.

HA_1_ 138, 186, 190, 194, 225, 226 and 228 had been previously shown to affect receptor binding to H1 HA [75], [76]. Among them, two residues, HA_1_ 190 and 225, play predominant roles in determining the receptor-binding specificity of H1 HA: D190/D225 for α(2,6) receptors in humans, D190/G225 for α(2,6) and α(2,3) receptors in swine, and E190/G225 for α(2,3) receptors in avian [11], [39], [40], [41], [42], [76]. Although changes at these two sites had been previously reported to cause antigenic drift in A(H1N1) epidemic strains [77], it was a somewhat common belief that key determinants of receptor-binding specificity are in general not subject to selection. Thus, the strong positive selection at HA_1_ 190 within subgroup I-*v* is quite unexpected.

### Positive Selection of Egg-Adapted Human A(H1N1) HAs during 1933∼1979

To compensate for the lack of non-egg-adapted human A(H1N1) HAs for the period of 1933∼1978, we separately collected a total of 32 different egg-adapted A(H1N1) HA sequences between 1933∼1979 that were free of sequence ambiguity (Fig. S2). These sequences as a group were analyzed by PAML 4.0, as well as two subgroups that covered the periods of 1947∼1957 (12 sequences) and 1948∼1979 (17 sequences) (Table 4, 5), keeping in mind of the egg-adapted mutations at HA_1_ 138, 144, 163, 189, 190, 225, and 226 [62], [63], [64]. The two subgroups 1947∼1957 and 1948∼1979 represented A(H1N1) viruses circulating in the 1950s and in the 1970s upon its reemergence in 1977, respectively. Given the close genetic and antigenic similarity of the reappeared A(H1N1) influenza virus in 1977 with the A(H1N1) viruses isolated in 1950 [22], [23], [24], it was of particular interest to investigate whether different evolutionary trends were adopted by the 1947∼1957 and 1948∼1979 subgroups.

**Table 4:** The values of log-likelihood (l), *d*_N_/*d*_S_, and parameter estimates in CODEML analysis of egg-adapted human A(H1N1) HAs between 1933-1979.

Model	l	*d*_N_/*d*_S_	Parameters estimates
**1933∼1979 (32 strains)** 1			
M0 (one-ratio)	−3336.01	0.411	ω = 0.411
M1a (nearly neutral)	−3283.75	0.336	*p*_0_ = 0.705 (*p*_1_ = 0.295), ω_0_ = 0.057 (ω_1_ = 1)
M2a (positive selection)	−3275.24	0.454	*p*_0_ = 0.721, *p*_1_ = 0.234 (*p*_2_ = 0.046), ω_0_ = 0.079 (ω_1_ = 1), ω_2_ = 3.571
M7 (beta)	−3285.56	0.345	*p* = 0.068, *q* = 0.129
M8a (beta&ω = 1)	−3283.78	0.336	*p*_0_ = 0.705 (*p*_1_ = 0.295), *p* = 6.100, *q* = 99, ω_s_ = 1
M8 (beta&ω>1)	−3275.41	0.452	*p*_0_ = 0.942 (*p*_1_ = 0.058), *p* = 0.206, *q* = 0.534, ω_s_ = 3.283
**1947∼1957 (12 strains)** 1			
M0 (one-ratio)	−2068.01	0.435	ω = 0.435
M1a (nearly neutral)	−2056.27	0.337	*p*_0_ = 0.689 (*p*_1_ = 0.311), ω_0_ = 0.038 (ω_1_ = 1)
M2a (positive selection)	−2046.27	0.501	*p*_0_ = 0.967, *p*_1_ = 0 (*p*_2_ = 0.033), ω_0_ = 0.256 (ω_1_ = 1), ω_2_ = 7.651
M7 (beta)	−2056.44	0.324	*p* = 0.012, *q* = 0.023
M8a (beta&ω = 1)	−2056.28	0.337	*p*_0_ = 0.688 (*p*_1_ = 0.312), *p* = 3.886, *q* = 99, ω_s_ = 1
M8 (beta&ω>1)	−2046.29	0.501	*p*_0_ = 0.967 (*p*_1_ = 0.033), *p* = 34.141, *q* = 99, ω_s_ = 7.667
**1948∼1979 (17 strains)** 1			
M0 (one-ratio)	−1759.58	0.385	ω = 0.385
M1a (nearly neutral)	−1751.46	0.260	*p*_0_ = 0.740 (*p*_1_ = 0.260), ω_0_ = 0 (ω_1_ = 1)
M2a (positive selection)	−1747.17	0.408	*p*_0_ = 0.794, *p*_1_ = 0.168 (*p*_2_ = 0.039), ω_0_ = 0 (ω_1_ = 1), ω_2_ = 6.226
M7 (beta)	−1751.61	0.300	*p* = 0.005, *q* = 0.012
M8a (beta&ω = 1)	−1751.46	0.260	*p*_0_ = 0.740 (*p*_1_ = 0.260), *p* = 0.005, *q* = 2.350, ω_s_ = 1
M8 (beta&ω>1)	−1747.19	0.411	*p*_0_ = 0.969 (*p*_1_ = 0.031), *p* = 0.006, *q* = 0.025, ω_s_ = 6.964

1 The analysis was performed on the first 337 residues of HA_1_ including the signal peptide.

**Table 5:** LRT tests and codons under positive selection for HA_1_ sequences of egg-adapted human A(H1N1) influenza viruses between 1933-1979.

			LRT (2Δl) (*p*-values)	Positively selected sites^1^
**1933∼1979 (32 strains)**	M2a	M2a-M1a	17.02 (0.0002)	**77 (95.8%)**, 225 (98.8%)
	M8	M8-M7	20.30 (0.0000)	**77 (98.7%)**, 225 (99.6%), **227 (97.7%)**
		M8-M8a	16.74 (0.0000)	
**1947∼1957 (12 strains)**	M2a	M2a-M1a	20.0 (0.0000)	**143 (99.3%), 264 (99.6%)**
	M8	M8-M7	20.30 (0.0000)	**143 (99.6%), 166 (95.1%), 264 (99.7%)**
		M8-M8a	19.98 (0.0000)	
**1948∼1979 (17 strains)**	M2a	M2a-M1a	8.58 (0.0137)	225 (99.1%)
	M8	M8-M7	8.84 (0.0120)	225 (99.8%)
		M8-M8a	8.54 (0.0017)	

1 Positively selected sites from PAML 4.0 [45] using Bayes Empirical Bayes analysis [72]. Only codons with greater than 95% posterior probabilities to be under positive selection were listed with the corresponding posterior probabilities shown in parentheses. Highlighted in bold were codons that were not associated with egg-adapted substitutions [62], [63], [64].

For both the entire group 1933∼1979 and the subgroup 1947∼1957, comparisons of M2a-M1a, M8-M7, or M8-M8a yielded large LRT values and very small *p*-values, suggesting the presence of positive selection at about 5% and 3% codons, respectively (Table 4, 5). However, it is noteworthy that the subgroup 1948∼1979 had much smaller LRT values, suggesting that the positive pressure of the entire group 1933∼1979 be mostly from the contribution of the subgroup 1947∼1957.

We further employed the CODEML in PAML 4.0 to analyze the positively selected codons in each group. The results were shown in Table 5 where highlighted in bold were the codons not known to be possible egg-adapted mutations (HA_1_ 138, 144, 163, 189, 190, 225, and 226) [62], [63], [64]. For the entire group 1933∼1979, HA_1_ 77, 225 and 227 were found to be under positive selection with greater than 95% posterior probability in model M8 (Fig. 1b). They were located in the antigenic sites Cb (HA_1_ 77) and Ca2 (HA_1_ 225 and 227), respectively (Fig. 1b). In addition, for the subgroup 1948∼1979, HA_1_ 225 was found to be under positive selection with greater than 99% posterior probability in both models M2a and M8. However, given the fact that these HAs were from egg-adapted A(H1N1) viruses in which HA_1_ 225 was one of the most frequently changed site [62], [63], [64], and the predominant residue at this site (Table 6), G225, was commonly found in swine and avian A(H1N1) HAs, it was possible that the changes at HA_1_ 225 was due to positive selection imposed by adaptation in eggs. At posterior probability of 90%, HA_1_ 138 and 189 were positively selected as well, however, both sites were involved in egg-adapted substitutions [62], [63], [64]. In sharp contrast, however, HA_1_ 143, 166 and 264 in the subgroup 1947∼1957 were found to be under positive selection (Table 5), none of which was among the previously identified egg-adapted mutations. Antigenically, these codons were located in the antigenic sites Ca2, Sa and Cb, respectively (Fig. 1b). For their relatively distant location from the receptor-binding site, HA_1_ 143, 166 and 264 are probably mutations driven by existing human immunity for antibody escape.

**Table 6:** Codons at HA_1_ 190 and 225 in human and swine A(H1N1) influenza viruses.

	D190	Non-D190	D225	G225	Non-D225/G225
Human 1979∼2008 Epidemic (575 sequences)	477 (83.0%)*	98 (17.0%)	565 (98.2%)	2 (0.4%)	8 (1.4%)
Human 1918 Pandemic (5 sequences)	5 (100%)	0	3 (60%)	2 (40%)	0
Human 2009 Pandemic (73 sequences)	73 (100%)	0	69 (94.5%)	1 (1.4%)	3 (4.1%)
Human 1947∼1957 (12 strains) (egg-adapted)	9 (75%)	3 (25%)	2 (16.7%)	10 (83.3%)	0
Human 1948∼1979 (17 strains) (egg-adapted)	16 (94.1%)	1 (5.9%)	4 (23.5%)	13 (76.5%)	0
Swine 1990∼2009 (42 sequences)	41 (97.6%)	1 (2.4%)	28 (66.6%)	12 (28.6%)	2 (4.8%)

* The number of cases that a particular type of residues occurs at each site. Shown in parenthesis was the occurrence in percentage.

Thus, there appeared to have different evolutionary patterns for the subgroup 1947∼1957 circulating in the 1950s and the subgroup 1948∼1979 circulating mostly in the 1970s. The former subgroup was subjected to positive selection pressure at HA_1_ 143, 166 and 264 (Table 5), and had a much larger variability at HA_1_ 190, with 25% being non-D190 (Table 6). In marked contrast, the latter subgroup was probably not under host-driven positive selection in humans and had highly conserved HA_1_ 190 (94.1% being D190).

### Evolution of Swine A(H1N1) HAs during 1990∼2009

Given the swine origin of the 2009 pandemic A(H1N1) HA, we also analyzed 42 non-redundant, non-ambiguous swine A(H1N1) HA sequences during 1990∼2009 available from GISAID/Epifludb (Table 7, Fig. S3). The reason that we focused on this period was mainly for the antigenic stasis of swine A(H1N1) until 1998 [8] since the introduction of the 1918 “Spanish” A(H1N1) virus into swine [1], [2], [3], [4], [5], [6], [7], [8]. Overall, the alternative models M2a and M8 fitted the data only marginally better than the null models M1a, M7 and M8a, respectively (Table 7). Thus, it seemed that swine A(H1N1) HAs during 1990∼2009 were not subjected to strong host-driven positive selection.

**Table 7:** The values of log-likelihood (l), *d*_N_/*d*_S_, and parameter estimates in CODEML analysis of swine A(H1N1) HAs between 1990–2009.

Model	l	*d*_N_/*d*_S_	Parameters estimates	LRT (2Δl) (*p*-values)
M0 (one-ratio)	−6021.08	0.158	ω = 0.158	
M1a (nearly neutral)	−5949.30	0.217	*p*_0_ = 0.864 (*p*_1_ = 0.136), ω_0_ = 0.094 (ω_1_ = 1)	LRT (M2a-M1a) = 1.24 (0.5379)
M2a (positive selection)	−5948.68	0.224	*p*_0_ = 0.864, *p*_1_ = 0.134 (*p*_2_ = 0.002), ω_0_ = 0.095 (ω_1_ = 1), ω_2_ = 3.682	
M7 (beta)	−5925.79	0.174	*P* = 0.381, *q* = 1.768	LRT (M8-M7) = 6.78 (0.0337) LRT (M8-M8a) = 3.40 (0.0326)
M8a (beta&ω = 1)	−5924.10	0.171	*p*_0_ = 0.970 (*p*_1_ = 0.030), *p* = 0.460, *q* = 2.617, ω_s_ = 1	
M8 (beta&ω)	−5922.40	0.177	*p*_0_ = 0.995 (*p*_1_ = 0.005), *p* = 0.413, *q* = 2.052, ω_s_ = 2.546	

The analysis was performed on the first 338 residues of HA_1_ including the signal peptide.

### Directional Evolution of Human A(H1N1) HAs

In order to test whether directional evolution of protein sequences existed in the evolution of human A(H1N1) HAs, we employed a maximum likelihood method developed by Kosakovsky Pond and colleagues [49]. In each subgroup, we used the oldest HA sequence as the root. In agreement with CODEML analysis reported in previous sections, among all non-egg adapted human A(H1N1) HAs, directional evolution was only identified in the subgroup I-*v*, at sites HA_1_ 143, 156, 158, 190, 193 and 197 (Table 8, 9). HA_1_ 143 belonged to the antigenic site Ca2 of A(H1N1) HA, whilst all other sites were located in the antigenic site Sb (Fig. 1b). Among these sites, HA_1_ 156, 190 and 193 were identified by CODEML in PAML 4.0 to be under positive selection with 99.7%, 100%, and 80.9% posterior probability in model M8, respectively (Table 3). In previous structural studies, residue HA_1_ 190 in 1934 human A(H1N1) HA and HA_1_ 190 and 193 in 1930 swine A(H1N1) HA were found to directly interact with bound human-like α(2,6)-receptors [78]. Thus, it remains to be investigated the impacts of directional evolution at HA_1_ 190 and 193 on receptor binding and antigenic drift.

**Table 8:** Directional selection analysis on human A(H1N1) Has.

	Tree L	Residue	*P*-Value	Bias	Proportion (%)	No. of Sites
Human I-*v* (100 sequences)	0.474	T	0.0002	32.995	5.4	2
		R	0.0002	12.583	11.8	1
		V	0.0004	34.478	4.0	2
		K	0.0023	29.301	7.2	1
Human 1933∼1979 (32 strains) (egg-adapted)	0.613	D	0.0000	78.148	3.4	1
Human 1948∼1979 (17 strains) (egg-adapted)	0.091	D	0.0007	133.611	5.1	1

**Table 9:** Sites found to be under directional selection in human A(H1N1) Has.

	Sites	Composition	Root	Preferred	Inferred Substitutions
Human I-*v* (100 sequences)	143	V_99_T_1_	V	V	T→_1_V
	156	G_90_R_9_E_1_	R	R	G→_1_E, G→_7_R
	158	N_96_K_4_	N	K	N→_4_K
	190	D_67_N_25_V_8_	D	V	D→_16_N, D→_4_V
	193	A_52_T_48_	A	T	A→_6_T
	197	T_82_K_18_	T	T	K→_2_T
Human 1933∼1979 (32 strains) (egg-adapted)	225	G_23_D_9_	D	D	D→_1_G, G→_6_D
Human 1948∼1979 (17 strains) (egg-adapted)	225	G_13_D_4_	G	D	G→_4_D

We also performed directional evolution study on egg-adapted human A(H1N1) HA sequences, and found that in both the entire group 1933∼1979 and the subgroup 1948∼1979, multiple favored mutations of D225→G and G225→D were detected (Table 8, 9). Given its involvement in egg-adaptation, the directional evolution at HA_1_ 225 may be the consequence of egg-adaptation. In contrast, no residues in the subgroup 1947∼1957 were identified to be under directional selection.

### Evolution of Human and Swine A(H1N1) HAs at HA_1_ 190 and 225

For their predominant roles in determining receptor-binding specificity of A(H1N1) HA, and the positive selection on HA_1_ 190 in the subgroup I-*v*, we further investigated the evolution of HA_1_ 190 and 225 in A(H1N1) strains during 1918∼1009. These included 653 non-egg-adapted HAs (five pandemic HAs from 1918∼1919, 575 epidemic HAs from 1979∼2008, and 73 pandemic HAs from 2009), and 42 swine HAs (Table 6). For the 575 epidemic HAs, HA_1_ 190 was highly variable (17.0% sequences did not have D190), while HA_1_ 225 was more conserved (only 1.8% sequences did not have D225) (Table 6). Among all the deviations (a total of 107 cases) from the ideal D190/D225 combination for human A(H1N1) viruses, two predominant ones were N190/D225 (69.2%) and V190/D225 (19.6%). At present, we don't know the exact effects of these mutations, or in combination with other concurring mutations at or around the receptor-binding site, on binding to human receptors. Further experiments are needed to clarify these issues. However, in previous studies, a single mutation D190N of A(H1N1) HA was shown to result in a lower binding affinity for human-like α(2,6) receptors, and a higher binding affinity for avian-like α(2,3) receptors [64].

The five HA sequences retrieved from victims of 1918 “Spanish” A(H1N1) influenza virus shared 98.9% to 99.8% sequence identity [11]. Among them, there were two non-synonymous substitutions of D225G, one in A/New York/1/1918 and the other one in A/London/1/1919 (Table 6). The HAs harboring the mutation D225G had reduced binding affinity for human receptors [11], [40], [41].

In the 73 HA sequences from the 2009 pandemic A(H1N1), D190 was strictly conserved, while D225 was 94.5% conserved (Table 6). At HA_1_ 225, the deviations were 1.1% for G225 and 3.3% for E225. Thus, the complete conservation at HA_1_ 190 and the nearly complete conservation at HA_1_ 225 were consistent to the importance of these residues in allowing for binding to human-like α(2,6) receptors [40], [41], supporting the substantially higher human-to-human transmissibility of the 2009 A(H1N1) virus than seasonal A(H1N1) viruses [5], [8].

Therefore, there were two distinct evolutionary trends in host-driven antigenic drift of human A(H1N1) HAs at residues in the receptor-binding site: the 1918 pandemic HAs underwent antigenic drift at HA_1_ 225, while the epidemic HAs undertook antigenic drift at HA_1_ 190. In the absence of selection, the 2009 A(H1N1) viruses were highly conserved at both HA_1_ 190 and 225, which was distinct from those two host-selected evolutionary trends (Table 6). With gradually established immunity among human population, we wondered how the 2009 A(H1N1) virus would undergo antigenic drift in the months to come. Thus, we also looked at the conservation at HA_1_ 190 and 225 in 42 swine A(H1N1) HA sequences (Table 6). Surprisingly, among these sequences, D190 was conserved at 97.6%, while D225 and G225 were observed at 66.6% and 28.6%, respectively. The similarly high variability of HA_1_ 225 in swine A(H1N1) HAs with that of 1918 pandemic HAs was consistent with the relative antigenic stasis of swine A(H1N1) until 1998 [8] and agreed well with the suggestion that the introduction of the 2009 pandemic A(H1N1) virus into humans be a single event or multiple events of similar viruses [1], [2], [3], [4], [5], [6], [7], [8].

The deviations from the ideal D190/D225 combination in A(H1N1) HAs might result in reduced binding to human receptors [11], [41], [42], [64], [79]. However, two possibilities, which are not mutually exclusive, may explain the fact that mutations are frequently observed at these two sites: one is that other concurring mutations at or around the receptor-binding site may sufficiently maintain the receptor binding affinity so that the overall binding affinity is largely unaffected; the second is that the gain in evading antibody neutralization far overweighs the reduction in receptor binding. Due to the overlapping locations of the ever-changing antigenic sites and the more-conserved receptor-binding site of HA, there is a constant dilemma of whether or not a residue at the receptor-binding site should change. Although the involvement of residues in antigenic drift that are critical for receptor binding was also observed in HAs of other types and subtypes including influenza B virus HA [80], H3 [43], [44]and H5 HA [44], [81], the interplay between these two opposing forces in HA evolution is still very poorly understood. Although previous studies on A(H3N2) HAs suggested covariation of antigenicity and receptor-binding specificity as a possible mechanism for the antigenic differences observed in viruses propagated in different cells [82], questions such as how residues involved in receptor binding are actively utilized for antigenic drift in influenza evolution in the same hosts need to be urgently addressed in order for us to comprehend the powerful strategies that the virus employs for recurring influenza infections.

### Implications for the 2009 Pandemic

By analyzing hundreds of A(H1N1) HA sequences between 1918∼2009, our study revealed positive selection in the subgroup I-*v* of A(H1N1) HAs. The positively selected codons were located at HA_1_ 156 and 190 in the Sb antigenic site [83]. It was surprising that HA_1_ 190, which is critical for receptor-binding specificity of A(H1N1) HAs, was also under positive selection. Through further analysis of HA_1_ 190, together with HA_1_ 225, the other critical determinant for receptor-binding specificity of A(H1N1), we found that the epidemic HAs and the 1918 pandemic and swine HAs favored one of these two sites for antigenic drift. Whether the 2009 pandemic A(H1N1) HA will adopt any of these two trends, or use a novel mechanism that does not involve HA_1_ 190 and 225, will unfold in the coming months. If the latter is to be used, the 2009 A(H1N1) viruses may maintain their intrinsic high transmissibility, which, together with mutations in other genes such as NS1 and PB1-F2 with signatures of elevated pathogenicity [1], [2], may suffice a new disastrous pandemic in the near future.

## Materials and Methods

### Phylogenetic Analysis of A(H1N1) HAs

We obtained all available HA sequences (over 1,000) of non-egg-adapted A(H1N1) viruses for the period of 1918∼2009 (as of July 10, 2009) from GISAID/Epifludb. We then removed the sequences with one or more ambiguous nucleotide sequences within the HA_1_ region and deleted identical sequences. This gave us a dataset of 652 HA sequences that included three 1918 pandemic HAs, 575 epidemic HAs from 1979∼2008 that collectively formed group I, and 73 pandemic HAs from 2009 and one HA from 2007 that belonged to group II. To facilitate the speed of computing, we further removed closely related sequences and obtained a dataset of 333 HA sequences. The program RDP3 (http://darwin.uvigo.es/rdp/rdp.html) [66] was used to make sure that no recombination was present in any of these HA sequences. The ClustalW method [68] with the MEGALIGN program of DNASTAR package (www.dnastar.com) was used for phylogenetic analysis of H1 HA sequences in the region of HA_1_ (Fig. S1).

Due to the historic use of eggs for amplification of influenza viruses before sequencing, there presented a vacuum in sequence for non-egg-adapted A(H1N1) viruses between 1919 and 1979. In order to gain insights into the evolution of A(H1N1) viruses for this period, we separately collected a total of 32 different egg-adapted A(H1N1) HA sequences between 1933∼1979 that were free of sequence ambiguity (Fig. S2). These sequences were similarly analyzed while keeping in mind of the possible egg-adapted mutations at HA_1_ 138, 144, 163, 189, 190, 225, and 226 [62], [63], [64].

In order to compare the evolution of swine A(H1N1) HA sequences, we also retrieved 42 unique swine H1 HA sequences for the period of 1990∼2009 that were free of ambiguous nucleotide sequences (Fig. S3). The reason that we focused on 1990∼2009 was that previous studies suggested that swine A(H1N1) viruses be antigenically stable for the period of 1930 to 1990s [84].

### Analysis of Positive Selection by PAML 4.0

The site-specific models implemented in the CODEML program in PAML 4.0 [45] was used to calculate heterogeneous selection pressure at amino-acid positions [45], [54], [72], [85]. The models used in this study were M0, M1a, M2a, M7 and M8. M1a (nearly neutral), M7 (beta) and M8a (beta and ω = 1) were null models that did not support ω>1. In contrast, the alternative models M2a (positive selection) and M8 (beta and ω), compared to M1a and M7 respectively, each had an additional class that allowed ω>1. Likelihood ratio tests (LRT) comparing M2a versus M1a, M8 versus M7, and M8 versus M8a provided test for the existence of positive selection. In the test, twice the log likelihood difference, 2Δl = 2(l_1_−l_0_), was calculated where l_1_and l_0_were the log likelihoods for the alternative model and null model, respectively. A larger value of LRT over those of χ^2^ distribution led to rejection of the null models [72]. In order to calculate the codon-substitution models for heterogeneous selection pressure at each codon, the Bayes Empirical Bayes (BEB) analysis implemented in CODEML [72] was used, which has been shown to yield robust results even for small datasets. For all calculations, multiple runs, each with different initial parameter values, were performed to ensure optimization and convergence.

### Directional Evolution of Protein Sequences Using HyPhy

Each group of A(H1N1) HA sequences aligned by the ClustalW method (Fig. S1, S2, S3) was input to the PhyML program [86] to generate an unrooted phylogenetic tree, which was then rooted using the Treeview software [87] by selecting the oldest sequence in each group as the root/ancestor. This rooted phylogenetic tree was used for directional evolution of protein sequences [49] implemented in the HyPhy [55] software package.

## Supporting Information

Figure S1 Phylogenetic tree of 333 HA sequences of A (H1N1) influenza viruses isolated between 1918∼2009 without egg-adaptation.
(41.64 MB TIF)

Figure S2 Phylogenetic tree of 32 HA sequences of egg-adapted human A(H1N1) influenza viruses isolated between 1933∼1979.
(1.02 MB TIF)

Figure S3 Phylogenetic tree of 42 HA sequences of swine A(H1N1) influenza viruses isolated between 1990∼2009.
(1.39 MB TIF)
