# Supplementary figures and images for: Evolutionary Trends of A(H1N1) Influenza Virus Hemagglutinin Since 1918
Source: PLoS One. 2009 Nov 17;4(11):e7789. doi: 10.1371/journal.pone.0007789 (PMC2773012; doi:10.1371/journal.pone.0007789)

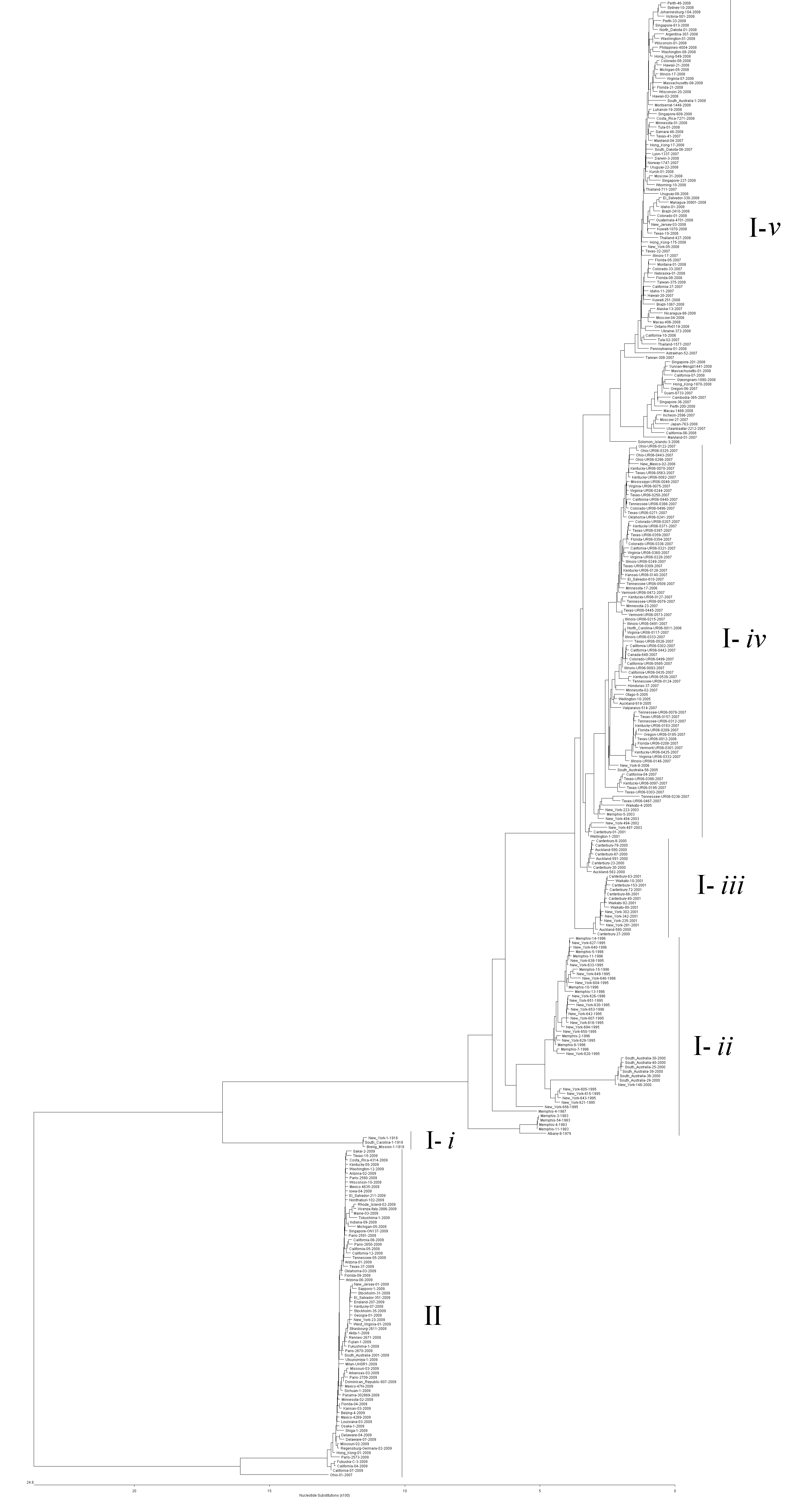

Supplement: Figure S1 — Phylogenetic tree of 333 HA sequences of A (H1N1) influenza viruses isolated between 1918∼2009 without egg-adaptation. (41.64 MB TIF) [file pone.0007789.s001.tif]

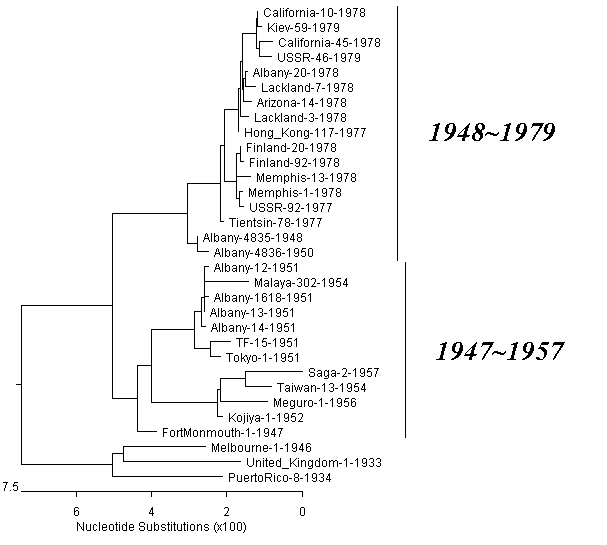

Supplement: Figure S2 — Phylogenetic tree of 32 HA sequences of egg-adapted human A(H1N1) influenza viruses isolated between 1933∼1979. (1.02 MB TIF) [file pone.0007789.s002.tif]

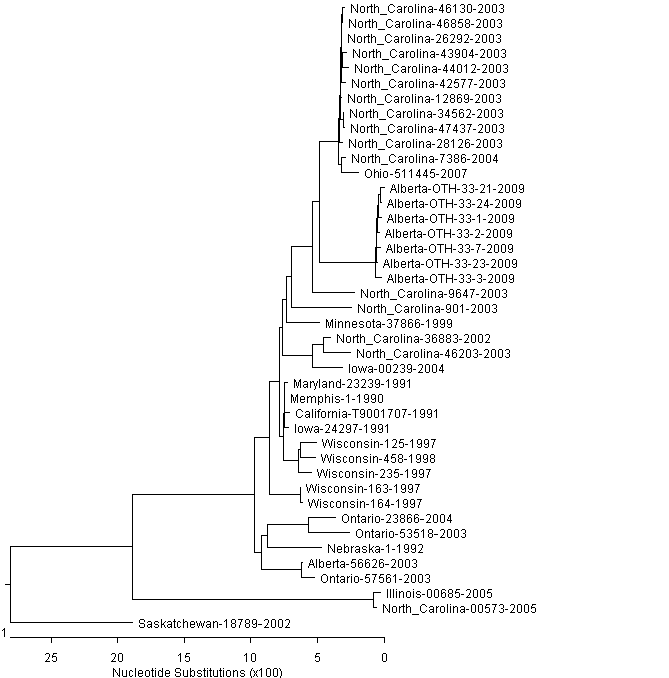

Supplement: Figure S3 — Phylogenetic tree of 42 HA sequences of swine A(H1N1) influenza viruses isolated between 1990∼2009. (1.39 MB TIF) [file pone.0007789.s003.tif]
